# Using Health Utility Index (HUI) for Measuring the Impact on Health-Related Quality of Life (HRQL) Among Individuals with Chronic Diseases

**DOI:** 10.1100/tsw.2004.128

**Published:** 2004-08-27

**Authors:** Frank Mo, Bernard C.K. Choi, Felix C.K. Li, Joav Merrick

**Affiliations:** ^1^ Centre for Chronic Disease Prevention and Control, Population and Public Health Branch, Health Canada, 120 Colonnade Road, Ottawa, Ontario, Canada K1A 0K9,; ^2^ National Institute of Child Health and Human Development, Office of the Medical Director, Division for Mental Retardation, Ministry of Social Affairs, Jerusalem and Zusman Child Development Center, Division of Pediatrics, Ben Gurion University of the Negev, Beer-Sheva, Israel

**Keywords:** quality of life, QOL, health-related QOL, Health Utilities Index (HUI), epidemiology, biostatistics, chronic illness, human development, public health, Canadian Community Health Survey, Canada

## Abstract

Quality of life is an important indicator in assessing the burden of disease, especially for chronic conditions. The Health Utilities Index (HUI) is a recently developed system for measuring the overall health status and health-related quality of life (HRQL) of individuals, clinical groups, and general populations. Using the HUI (constructed based on eight attributes: vision, hearing, speech, mobility, dexterity, cognition, emotion, and pain/discomfort) to measure the HRQL for chronic disease patients and to detect possible associations between HUI system and various chronic conditions, this study provides information to improve the management of chronic diseases.This study is of interest to data analysts, policy makers, and public health practitioners involved in descriptive clinical studies, clinical trials, program evaluation, population health planning, and assessments. Based on the Canadian Community Health Survey (CCHS) for 2000–01, the HUI was used to measure the quality of life for individuals living with various chronic conditions (Alzheimer/other dementia, effects of stroke, urinary incontinence, arthritis/rheumatism, bowel disorder, cataracts, back problems, stomach/intestinal ulcers, emphysema/COPD, chronic bronchitis, epilepsy, heart disease, diabetes, migraine headaches, glaucoma, asthma, fibromyalgia, cancers, high blood pressure, multiple sclerosis, thyroid condition, and other remaining chronic diseases). Logistic Regression Model was employed to estimate the associations between the overall HUI scores and various chronic conditions. The HUI scores ranged from 0.00 (corresponding to a state close to death) to 1.00 (corresponding to perfect health); negative scores reflect health states considered worse than death. The mean HUI score by sex and age group indicated the typical quality of life for persons with various chronic conditions. Logistic Regression results showed a strong relationship between low HUI scores (≤ 0.5 and 0.06–1.0) and certain chronic conditions. Age- and sex-adjusted Odds Ratio (OR) and *p* values showed an effect among individuals diagnosed with each chronic disease on the overall HUI score. Results of this study showed that arthritis/rheumatism, heart disease, high blood pressure, cataracts, and diabetes had a severe impact on HRQL. Urinary incontinence, Alzheimer/other dementia, effects of stroke, cancers, thyroid condition, and back problems have a moderate impact. Food allergy, allergy other than food, asthma, migraine headaches, and other remaining chronic diseases have a relatively mild effect. It is concluded that major chronic diseases with significant health burden were associated with poor HRQL. The HUI scores facilitate the measurement and interpretation of results of health burden and the HRQL for individuals with chronic diseases and can be useful for development of strategies for the prevention and control of chronic diseases.

## INTRODUCTION

According to Ventegodt et al.[1], the quality of life is how good a life each individual feels he or she has. Each individual personally evaluates how he or she views things and his or her feelings and notions. Whether an individual is content with life and happy are aspects that reflect the subjective quality of life[1].

With evolution from a focus on reducing mortality to reducing disability due to chronic diseases, there is a need for methods of measuring the effect of chronic diseases on health status, which is more complicated than simply measuring how often the diseases cause death[2]. Therefore, developing valid and reliable methods for assessing the relative impact and distinguishing between chronic diseases is important when establishing program priorities and for estimating the cost and burden that various diseases present[3]. Quality of life is an important indicator in assessing the burden of disease especially for chronic conditions.

The Health Utilities Index (HUI) is based on the Comprehensive Health Status Measurement System (CHSMS) and a generic multiattribute preference-based measure of health status and health-related quality of life (HRQL) that is widely used as an outcome measure in clinical studies, in population health surveys, in the estimation of quality-adjusted life years, and in economic evaluations[4]. It provides a description of an individual’s overall functional health based on eight attributes: vision, hearing, speech, mobility (ability to get around), dexterity (use of hands and fingers), cognition (memory and thinking), emotion (feelings), and pain/discomfort[5].

The HUI is able to synthesize both quantitative and qualitative aspects of health. It is a family of generic health status and HRQL measures, including the HUI Mark 1 (HUI1), Mark 2 (HUI2), and Mark 3 (HUI3), which are frequently used both in clinical and population health studies in many countries, including Canada[6]. The HUI3 has two components: (1) the health status classification system and (2) the preference-based scoring system[7,8].

In addition, in describing the functional health status levels, the HUI3 has a single numerical value for any possible combination of levels of the eight self-reported health attributes. The HUI3 maps any one of the vectors of the eight health attribute levels into a summary health value between –0.36 and 1.00. On that scale, the most preferred health level (perfect health) is rated 1.00 and death is rated 0.00, while negative scores reflect health states considered worse than death. The HUI3 was developed by McMaster University’s Centre for Health Economics and Policy Analysis and is derived using societal preferences from a random sample of 500 people within the boundaries of the City of Hamilton-Wentworth, Ontario, Canada[9]. The scores of the HUI embody the views of society concerning health status. These views are termed societal preferences, since preferences about various health states are elicited from a representative sample of individuals.

This project is of interest to analysts, policy makers, and decision makers involved with descriptive clinical studies, clinical trials, programed evaluations, measuring population health, and planning assessments. To use the HUI to measure the quality of life for the individuals with poorer HRQL than the general population and to identify the possibility of associations between HUI system and various chronic conditions may provide information to improve the management of quality of life for chronic diseases in Canada.

## METHODS

The study was based on the Canadian Community Health Survey (CCHS) for 2000–01[10], which was conducted by Statistics Canada, Health Canada, and the Canadian Institute for Health Information (CIHI) to provide regular and timely cross-sectional estimates of health determinants, health status, and health system utilization for 136 health regions across the country, with a total sample of 130,880. The target population of the CCHS included all household residents 12 years of age and over in all provinces and territories; with the principal exclusion of populations on Indian Reserves, Canadian Forces Bases, and some remote areas. As with other Statistics Canada surveys, the results of this survey will aid in the development of public policy, help in the understanding of the determinants of health, and increase understanding of the relationship between health status and health care utilization. This method was used to estimate graphical and adjusted distributions of the prevalence for various chronic diseases and the HUI3 scores, in order to measure the quality of life for individuals living with various chronic conditions (diabetes, heart disease, high blood pressure, asthma, COPD, stroke, cataracts, cancer, glaucoma, and renal diseases). Logistic Regression Model was employed in this study to estimate the associated effects between the overall HUI3 scores ($\leq 0.5$ and 0.6–1.0) and various predictive factors for the individuals with chronic disease by controlling for age (12–39, 40–64, and 65+) and sex (female as reference)**.** The adjusted Odds Ratio (OR) and $p$ values were also calculated.

## RESULTS

The scores of HUI ranged from 0.00 (corresponding with state close to death) to 1.00 (corresponding with perfect health). The mean HUI score by sex and age group indicated the typical quality of life for people with various chronic conditions. Table 1 shows the distribution and correlated effects between the mean HUI scores and the selected chronic conditions. High HUI was found for allergy other than food, food allergy, and migraine headaches.

Fig. 1 and Table 2 indicate the general relative impact on HRQL of selected chronic diseases. Arthritis/rheumatism, heart disease, high blood pressure, cataracts, and diabetes showed the greatest impact on HRQL than other chronic diseases. There are higher impacts on HRQL for females than males in arthritis/rheumatism, fibromyalgia, bowel disorder, cataracts, glaucoma, and thyroid condition. By contrast, male individuals with diabetes, heart attack, and allergy other than food, had higher impacts on HRQL than females.

Our study also estimated the impact of various chronic diseases by four age groups (12–39, 40–64, and 65+). In the 12–39 age group, urinary incontinence, epilepsy, back problems, arthritis/rheumatism, and bowel disorder had the severe effects on HRQL. However, among the 40–64 age group and aged 65 years and over group, Alzheimer/other dementia, effects of stroke, cataracts, Parkinson’s disease, diabetes, and chronic bronchitis had high impact than younger age groups (Fig. 2).

**TABLE 1**
**Number of Cases, Percent, and Correlation Coefficient with HUI3 Scores of Selected Chronic Conditions by Gender, Household Population Aged 12 and Over**

| Chronic Diseases | Males | | Females | | Total | | Adjusted Correlation Coefficient with HUI3† | | | Unadjusted Correlation Coefficient with HUI3 |
|---|---|---|---|---|---|---|---|---|---|---|
| | Cases* | %* | Cases* | %* | Cases* | %* | All Ages | Male | Female | All Ages |
| Allergy other than food | 12,009 | 10.26 | 20,754 | 14.97 | 32,763 | 12.62 | 0.07 | 0.08 | 0.02 | 0.05 |
| Asthma | 6756 | 5.03 | 4139 | 3.42 | 10,895 | 4.22 | 0.06 | 0.03 | 0.02 | 0.06 |
| Migraine headaches | 3051 | 2.44 | 9040 | 6.69 | 12,091 | 4.57 | 0.05 | 0.03 | 0.04 | 0.10 |
| Food allergy | 3250 | 2.71 | 6560 | 4.57 | 9810 | 3.64 | 0.01 | 0.02 | 0.01 | 0.05 |
| Epilepsy | 440 | 0.29 | 370 | 0.28 | 810 | 0.29 | 0.01 | 0.01 | 0.01 | 0.05 |
| Multiple sclerosis | 227 | 0.17 | 94 | 0.08 | 321 | 0.13 | –0.01 | **–0.01** | **–0.01** | 0.05 |
| Chronic fatigue syndrome | 757 | 0.56 | 277 | 0.22 | 1034 | 0.39 | –0.02 | –0.01 | –0.02 | 0.11 |
| Fibromyalgia | 219 | 0.16 | 1479 | 0.92 | 1698 | 0.54 | –0.05 | –0.02 | –0.05 | 0.12 |
| Heart attack | 1368 | 15.33 | 1967 | 26.74 | 3335 | 21.04 | –0.05 | **–0.04** | **–0.03** | 0.06 |
| Bowel disorder | 883 | 0.67 | 2274 | 1.41 | 3157 | 1.04 | –0.06 | –0.03 | –0.06 | 0.10 |
| Parkinson's disease | 149 | 0.10 | 129 | 0.12 | 278 | 0.11 | –0.06 | –0.04 | –0.04 | 0.05 |
| Angina | 1675 | 19.86 | 1341 | 17.79 | 3016 | 18.83 | –0.07 | –0.06 | –0.07 | 0.18 |
| Alzheimer/other dementia | 162 | 0.14 | 157 | 0.12 | 319 | 0.13 | **–0.07** | **–0.04** | **–0.07** | 0.07 |
| Chronic bronchitis | 2691 | 1.75 | 1364 | 1.06 | 4055 | 1.41 | –0.07 | –0.04 | –0.05 | 0.10 |
| Other chronic condition | 8438 | 5.25 | 6045 | 5.04 | 14,483 | 5.65 | –0.07 | –0.04 | –0.05 | 0.16 |
| Stomach/intestinal ulcers | 2015 | 1.36 | 2555 | 1.57 | 4570 | 1.47 | –0.07 | –0.05 | –0.05 | 0.12 |
| Congestive heart failure | 922 | 11.52 | 691 | 9.43 | 1613 | 10.48 | –0.08 | –0.08 | –0.10 | 0.20 |
| Emphysema/COPD | 653 | 0.51 | 722 | 0.72 | 1375 | 0.62 | –0.11 | –0.09 | –0.08 | 0.09 |
| Back problem | 10,839 | 8.27 | 13,654 | 9.26 | 24,493 | 8.77 | –0.12 | –0.06 | –0.09 | 0.23 |
| Effects of stroke | 757 | 0.51 | 848 | 0.51 | 1605 | 0.51 | –0.13 | –0.09 | –0.10 | 0.11 |
| Cancer | 1453 | 0.93 | 1143 | 0.83 | 2596 | 0.88 | –0.14 | –0.10 | –0.09 | 0.08 |
| Glaucoma | 762 | 0.58 | 1381 | 0.87 | 2143 | 0.73 | –0.15 | –0.08 | –0.13 | 0.08 |
| Urinary incontinence | 2551 | 1.51 | 979 | 0.70 | 3530 | 1.11 | –0.16 | –0.09 | –0.13 | 0.16 |
| Thyroid condition | 1092 | 0.79 | 6082 | 3.97 | 7174 | 2.38 | –0.17 | –0.07 | –0.15 | 0.09 |
| Diabetes | 3102 | 1.98 | 2972 | 2.17 | 6074 | 2.06 | –0.20 | –0.16 | –0.14 | 0.12 |
| Heart disease | 3864 | 2.64 | 4104 | 2.35 | 7968 | 2.50 | –0.28 | –0.18 | –0.20 | 0.16 |
| Cataracts | 2071 | 1.61 | 4330 | 2.70 | 6401 | 2.16 | –0.31 | –0.16 | –0.25 | 0.15 |
| High blood pressure | 11,092 | 7.10 | 7443 | 5.64 | 18,535 | 6.37 | –0.38 | –0.20 | –0.31 | 0.18 |
| Arthritis/rheumatism | 8460 | 5.61 | 16051 | 9.62 | 24511 | 7.62 | –0.40 | –0.20 | –0.32 | 0.30 |

* Cases unweighted, percent weighted.
† Adjusted correlation coefficient with HUI3 by age and sex.

Canadian Community Health Survey (CCHS), Canada, 2000–01.

Results from the Logistic Regression Model showed the different adjusted OR and *p* values between the overall HUI3 scores (≤ 0.5 and 0.6–1.0) and various predictive factors for the individuals with chronic disease positively related to almost chronic conditions. The highest impacts on HRQL are from Alzheimer/other dementia (OR = 9.96, $p$ = 0.001), effects of stroke (OR = 9.96, $p$ = 0.001), multiple sclerosis (OR = 9.20, $p$ = 0.001), Parkinson's disease (OR = 8.28, $p$ = 0.001), chronic fatigue syndrome (OR = 7.41, $p$ = 0.001), heart diseases (OR = 6.33, $p$=0.001), emphysema/COPD (OR = 5.51, $p$ = 0.001), and urinary incontinence (OR = 4.71, $p$ = 0.001).

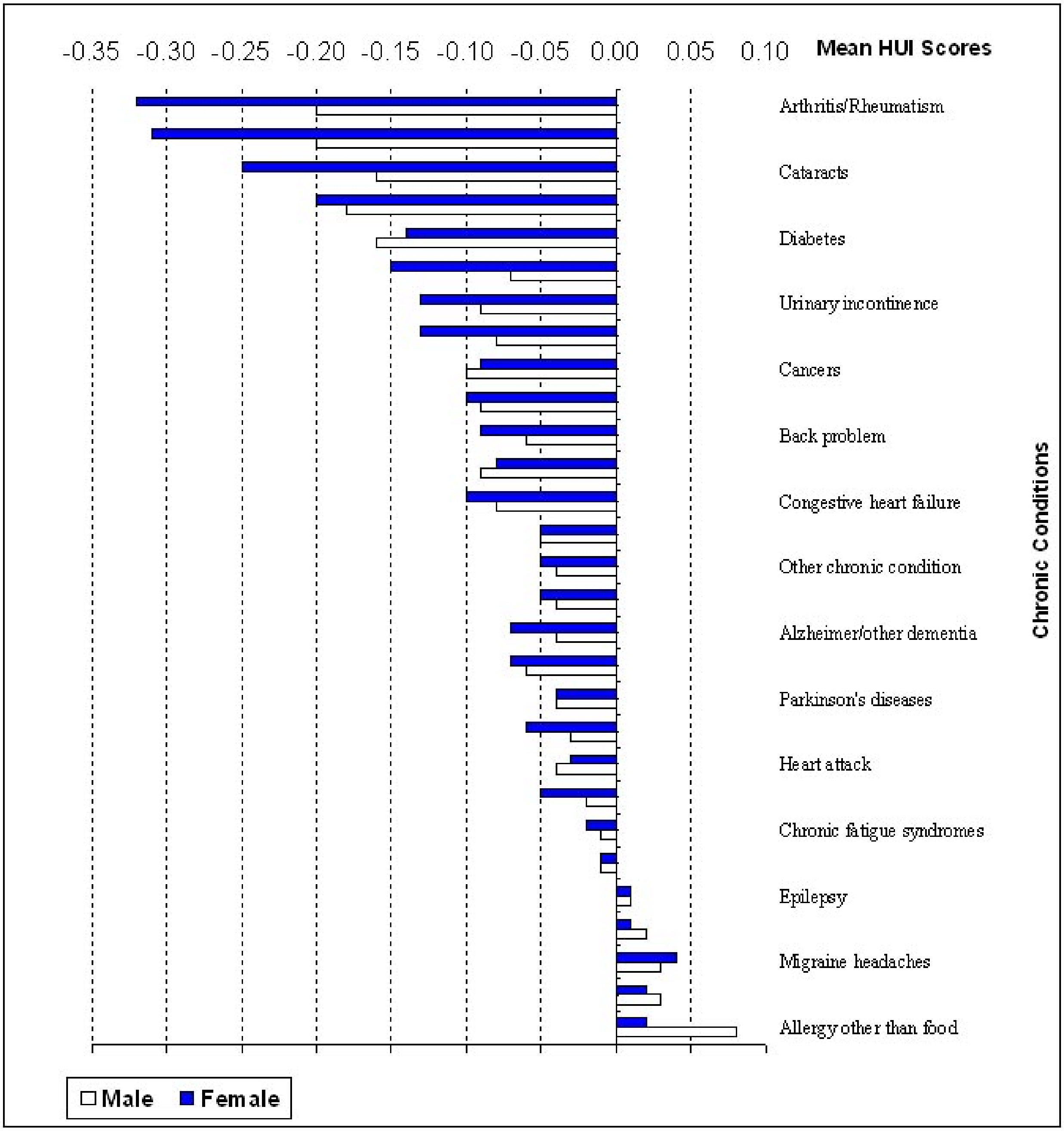

**FIGURE 1.** Impact of selected chronic conditions on HRQL by gender and household population aged 12 and over. CCHS Data, 2000–01.

The impact on HRQL of some chronic conditions appear to be greater among those aged 65 and over, compared to those in younger age groups. The OR and *p* values were relatively high for effects of stroke (OR = 4.89, *p* = 0.001), emphysema/COPD (OR = 3.19, *p* = 0.001), Alzheimer/other dementia (OR = 2.30, *p* = 0.001), Parkinson's disease (OR = 2.18, *p* = 0.001), cataracts (OR = 2.14, *p* = 0.001), chronic bronchitis (OR = 2.05, *p* = 0.001), high blood pressure (OR = 1.83, *p* = 0.001), and diabetes (OR = 1.69, *p* = 0.001). However, in contrast, there was high impact on HRQL for the younger age group (12–39 years) of urinary inconvenience, arthritis/rheumatism, food allergy, epilepsy, and back problems. Comparing the sexes, the impact on quality of life for males was relatively high for chronic fatigue syndrome (OR = 14.50, *p* = 0.001), multiple sclerosis (OR = 10.96, *p* = 0.001), diabetes (OR = 4.87, *p* = 0.001), and asthma (OR = 1.70, *p*=0.001) (Tables 2, 3).

**TABLE 2**
**Adjusted OR for HUI ≤ 0.5 and *p* Values from Logistic Regression Model Between HUI Scores and the Selected Chronic Conditions by Gender**

| Chronic Diseases | HUI[1] Score | | Females | | Males | |
|---|---|---|---|---|---|---|
| | OR[2] | *p* | OR[2] | *p* | OR[2] | *p* |
| Food allergy | 1.34 | 0.001 | 1.50 | 0.001 | 1.46 | 0.001 |
| Asthma | 1.42 | 0.001 | 1.52 | 0.001 | 1.70 | 0.001 |
| Fibromyalgia | 3.85 | 0.001 | 9.64 | 0.001 | 8.76 | 0.001 |
| Arthritis/rheumatism | 4.68 | 0.001 | 5.18 | 0.001 | 4.38 | 0.001 |
| Back problem | 3.41 | 0.001 | 3.54 | 0.001 | 3.29 | 0.001 |
| High blood pressure | 2.58 | 0.001 | 2.44 | 0.001 | 2.67 | 0.001 |
| Migraine headaches | 2.37 | 0.001 | 3.23 | 0.001 | 2.03 | 0.001 |
| Diabetes | 3.27 | 0.001 | 2.93 | 0.001 | 4.87 | 0.001 |
| Epilepsy | 3.73 | 0.001 | 3.09 | 0.001 | 4.08 | 0.001 |
| Heart disease | 6.33 | 0.001 | 4.05 | 0.001 | 4.96 | 0.001 |
| Cancer | 3.46 | 0.001 | 3.79 | 0.001 | 3.17 | 0.001 |
| Stomach/intestinal ulcers | 3.92 | 0.001 | 4.45 | 0.001 | 4.70 | 0.001 |
| Effects of stroke | 9.96 | 0.001 | 9.75 | 0.001 | 10.02 | 0.001 |
| Urinary incontinence | 4.71 | 0.001 | 9.64 | 0.001 | 7.78 | 0.001 |
| Bowel disorder | 3.97 | 0.001 | 3.76 | 0.001 | 2.62 | 0.001 |
| Alzheimers/other dementia | 9.66 | 0.001 | 7.32 | 0.001 | 3.12 | 0.001 |
| Cataracts | 3.76 | 0.001 | 3.41 | 0.001 | 2.61 | 0.001 |
| Glaucoma | 3.31 | 0.001 | 3.21 | 0.001 | 2.93 | 0.001 |
| Thyroid condition | 2.22 | 0.001 | 2.11 | 0.001 | 1.99 | 0.001 |
| Parkinson's disease | 8.28 | 0.001 | 9.08 | 0.001 | 8.54 | 0.001 |
| Multiple sclerosis | 9.20 | 0.001 | 6.66 | 0.001 | 10.95 | 0.001 |
| Chronic fatigue syndrome | 7.41 | 0.001 | 9.82 | 0.001 | 14.50 | 0.001 |
| Heart attack | 1.34 | 0.001 | 1.25 | 0.003 | 1.64 | 0.001 |
| Angina | 1.90 | 0.001 | 1.48 | 0.001 | 1.70 | 0.001 |
| Congestive heart failure | 2.75 | 0.001 | 3.07 | 0.001 | 2.40 | 0.001 |
| Chronic bronchitis | 3.35 | 0.001 | 3.68 | 0.001 | 3.05 | 0.001 |
| Emphysema/COPD | 5.51 | 0.001 | 6.33 | 0.001 | 4.83 | 0.001 |

[1] HUI scores in two categories: ≤ 0.5, and 0.6–1.0.
[2] OR adjusted for age and sex.

Canadian Community Health Survey (CCHS), Canada, 2000–01.

## DISCUSSION

Population-based health surveys are useful for estimating the long-term consequences of eliminating chronic diseases in various cohorts, as they directly estimate the health status of a population at a defined time period such as the National Population Health Survey (NPHS)[11] and CCHS[10] in Canada. These estimations of cause-specific health status are the combined impact on HRQL of disease incidence, prevalence, duration, and severity[12].

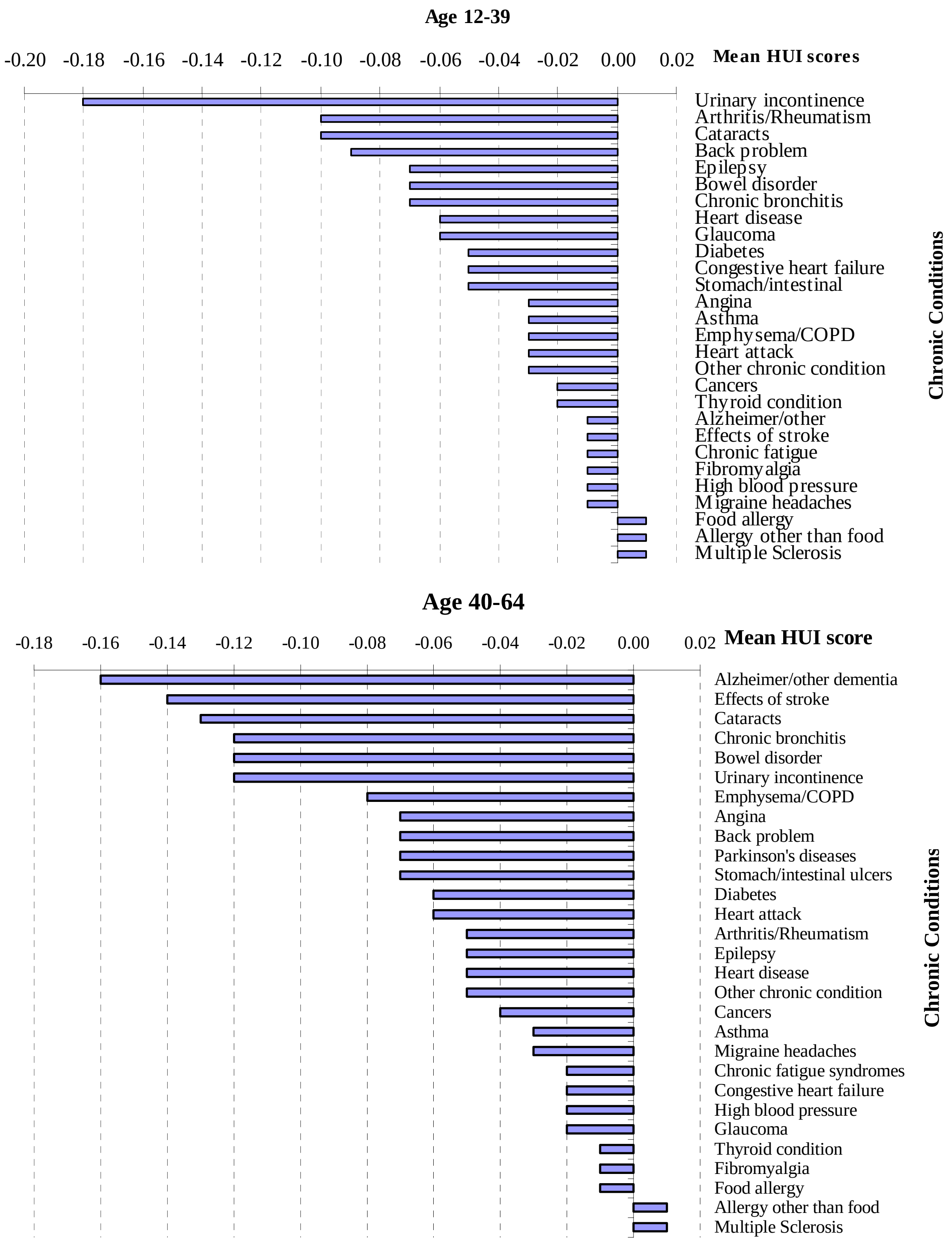

**FIGURE 2**. Impact of selected chronic conditions on HRQL by age group and household population aged 12 and over. CCHS Data, 2000–01.

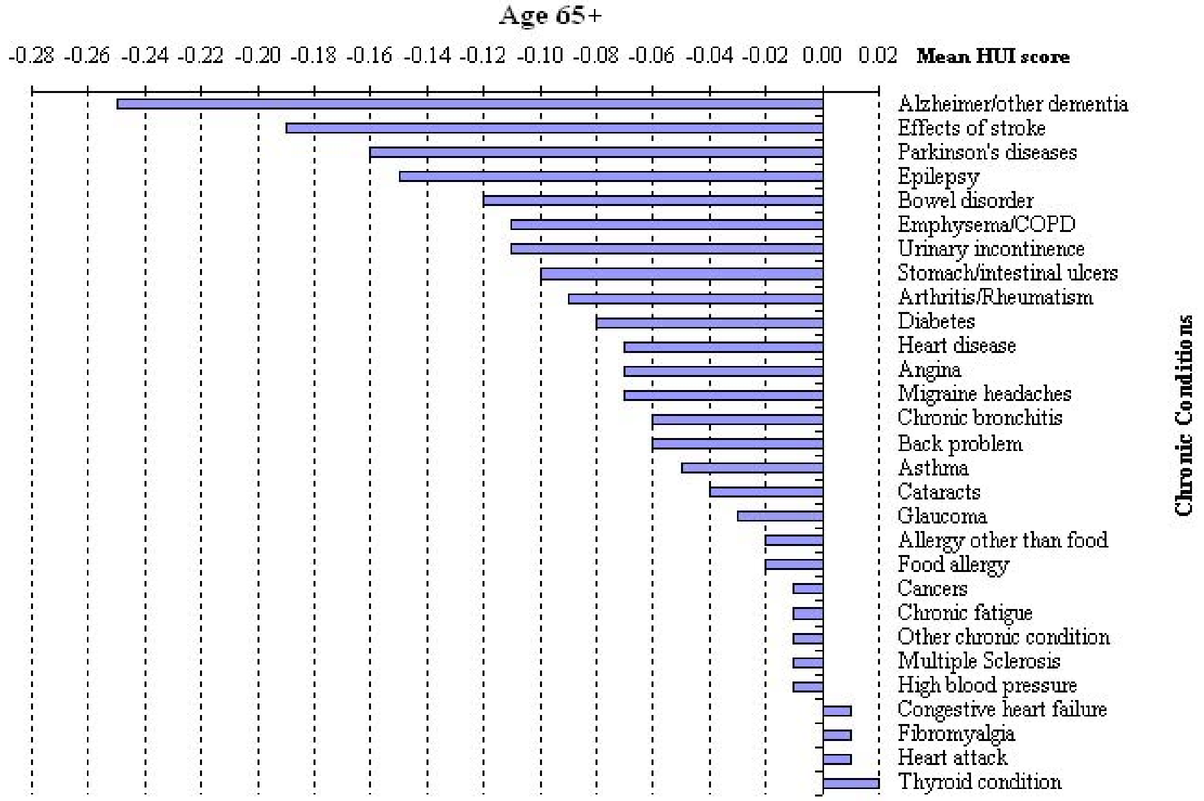

**FIGURE 2 (continued).**

The HUI3 is a psychometric instrument developed to assign utilities to patients' health states and a multidimensional, preference-based measure of health status and HRQL. HUI3 scores correlate strongly with self-ratings of health status and functional disability and vary according to age, gender, and occupation. In comparative studies relating to HRQL, it is necessary to carry out adjusted comparison of the health status of the different groups[13]. Stolk and Busschbach confirmed that disease-specific utility scores could be used in HRQL analysis by converting them to a generic scale[14].

Ruiz et al. used the HUI3 Spanish version to validate a modelling sample to develop the Multi Attribute Utility Function (MAUF) and a directed measure sample to validate the MAUF. Their results showed that the HUI3 Spanish version is feasible and reliable with a good test-retest correlation (0.909, $p < 0.001$). Convergent validity is good with EuroQoL (r = 0.788–0.793), but the Spanish and Canadian utility function differed significantly ($p < 0.001$)[15].

In another study, Taylor et al.[16] reported that the majority of children admitted to a pediatric intensive care unit survive with an excellent functional outcome and quality of life. Although 16.4% had an unfavorable quality of life, 83.6% of the children survived with a favorable quality of life. Long-term outcome assessment provides a basis for observing trends in outcome over time within the same institution.

**TABLE 3**
**Adjusted OR for HUI ≤ 0.5 and *p* Values from Logistic Regression Model Between HUI Scores and the Selected Chronic Conditions by Age**

| Chronic Diseases | HUI[1] Score | | 12–39 years | | 40–64 years | | 65+ years | |
|---|---|---|---|---|---|---|---|---|
| | OR[2] | *p* | OR[2] | *p* | OR[2] | *p* | OR[2] | *p* |
| Food allergy | 1.34 | 0.001 | 1.76 | 0.001 | 1.54 | 0.001 | 1.41 | 0.001 |
| Asthma | 1.42 | 0.001 | 1.69 | 0.001 | 1.96 | 0.001 | 1.70 | 0.001 |
| Fibromyalgia | 3.85 | 0.001 | 2.55 | 0.001 | 4.35 | 0.001 | 2.75 | 0.001 |
| Arthritis/rheumatism | 4.68 | 0.001 | 4.22 | 0.001 | 4.01 | 0.001 | 2.48 | 0.001 |
| Back problem | 3.41 | 0.001 | 3.18 | 0.001 | 3.86 | 0.001 | 2.41 | 0.001 |
| High blood pressure | 2.58 | 0.001 | 1.01 | 0.058 | 1.58 | 0.001 | 1.83 | 0.001 |
| Migraine headaches | 2.37 | 0.001 | 1.48 | 0.001 | 2.86 | 0.001 | 1.76 | 0.001 |
| Diabetes | 3.27 | 0.001 | 1.07 | 0.046 | 1.26 | 0.008 | 1.69 | 0.001 |
| Epilepsy | 3.73 | 0.001 | 1.65 | 0.001 | 2.20 | 0.001 | 1.15 | 0.005 |
| Heart disease | 6.33 | 0.001 | 1.08 | 0.043 | 3.06 | 0.001 | 2.42 | 0.001 |
| Cancer | 3.46 | 0.001 | 1.14 | 0.018 | 2.08 | 0.001 | 1.79 | 0.001 |
| Stomach/intestinal ulcers | 3.92 | 0.001 | 1.04 | 0.041 | 1.91 | 0.001 | 2.67 | 0.001 |
| Effects of stroke | 9.96 | 0.001 | 0.82 | 0.089 | 1.65 | 0.001 | 4.89 | 0.001 |
| Urinary incontinence | 4.71 | 0.001 | 2.81 | 0.001 | 2.26 | 0.001 | 1.55 | 0.001 |
| Bowel disorder | 3.97 | 0.001 | 2.86 | 0.001 | 3.58 | 0.001 | 1.88 | 0.001 |
| Alzheimers/other dementia | 9.66 | 0.001 | 0.00 | 0.000 | 1.82 | 0.001 | 2.30 | 0.001 |
| Cataracts | 3.76 | 0.001 | 0.99 | 0.086 | 1.80 | 0.001 | 2.14 | 0.001 |
| Glaucoma | 3.31 | 0.001 | 1.03 | 0.049 | 1.40 | 0.001 | 1.64 | 0.001 |
| Thyroid condition | 2.22 | 0.001 | 1.29 | 0.001 | 1.65 | 0.001 | 1.46 | 0.001 |
| Parkinson's disease | 8.28 | 0.001 | 0.96 | 0.079 | 1.88 | 0.001 | 2.18 | 0.001 |
| Multiple sclerosis | 9.20 | 0.001 | 1.07 | 0.048 | 1.49 | 0.001 | 1.20 | 0.001 |
| Chronic fatigue syndrome | 7.41 | 0.001 | 1.29 | 0.001 | 3.26 | 0.001 | 2.47 | 0.001 |
| Heart attack | 1.34 | 0.001 | 0.92 | 0.069 | 1.62 | 0.001 | 1.21 | 0.001 |
| Angina | 1.90 | 0.001 | 1.03 | 0.049 | 2.14 | 0.001 | 1.61 | 0.001 |
| Congestive heart failure | 2.75 | 0.001 | 1.25 | 0.001 | 2.85 | 0.001 | 2.62 | 0.001 |
| Chronic bronchitis | 3.35 | 0.001 | 1.07 | 0.044 | 1.97 | 0.001 | 2.05 | 0.001 |
| Emphysema/COPD | 5.51 | 0.001 | 0.99 | 0.055 | 2.59 | 0.001 | 3.19 | 0.001 |

1 HUI scores in two categories: ≤ 0.5, and 0.6–1.0.
2 OR adjusted for age and sex.

Canadian Community Health Survey (CCHS), Canada, 2000–01.

In a study to estimate the burden of chronic conditions and to measure the HRQL in Ontario, Canada, the results of HRQL varied from one condition to another[12]. Ontario men reporting disabling injuries had a mean HUI of 0.77, while those with respiratory disorders had a mean HUI of 0.94. Age has an independent effect on the HUI and age alone is not sufficient to explain the differences in mean HUI between chronic conditions. The changes in the HUI after adjusting for age suggest not only that there is an age effect, but also that it differs across chronic conditions. It is similar to our results in the Logistic Regression analysis. This demonstrated that a polytomous index (like the HUI) for different chronic conditions and ages and sexes might more accurately assess a utility value for HRQL. Similar to previous studies[17,18,19,20,21], the Multivariate Logistical Regression analysis in our study showed that effects of stroke (OR = 4.89, $p$ = 0.001), emphysema/COPD (OR = 3.19, $p$ = 0.001), Alzheimer/other dementia (OR = 2.30, $p$ = 0.001), Parkinson's disease (OR = 2.18, $p$ = 0.001), cataracts (OR = 2.14, $p$ = 0.001), chronic bronchitis (OR = 2.05, $p$ = 0.001), high blood pressure (OR = 1.83, $p$ = 0.001), and diabetes (OR = 1.69, $p$ = 0.001) have the greatest impact on HRQL in the 65 years and over age groups, because of their duration of damage. This corresponds to the Neumann et al.[22] study, where their results in multiple regression analyses indicated that the Alzheimer's disease stage was a negative and significant predictor of utility scores for patients. However, in this aged group, the urinary inconvenience, arthritis/rheumatism, food allergy, and epilepsy were less impactful on HRQL than at aged 12–39 groups. Our results are also similar to Schultz and Kopec reports[2].

Among other chronic conditions, obesity also has a significant impact on both quality of life and health. Trakas and colleagues[23] measured the clinically relevant difference in the health state utilities of obese and nonobese individuals in Canada, by using the HUI3 measurement method. The prevalence of obesity (BMI ≥ 30 kg/m$^2$) in this Canadian population was 13.3%. The average difference in HUI3 scores between normal weight and morbidly obese respondents was 0.04 ($p < 0.001$). Statistically significant differences across BMI categories were found in each of the eight component attributes of the HUI3 ($p < 0.05$). The most differences between normal and obese patients were found in cognition, mobility, and pain. There were twofold or more increases in the proportion of individuals in poorer classifications of health when normal weight respondents were compared with the morbidly obese.

The results in the Boyle et al. study[24] indicated the HUI scores in the Canadian General Social Survey that Kappa estimates of test-retest reliability for individual questions varied from 0.184–0.766, the eight attributes were estimated from 0.137–0.728, and provisional index scores of 0.767 were also obtained. The only two exceptions to this were limitations in speech and dexterity, which were reported very infrequently.

This study has several limitations including temporality and self-report. The design of the CCHS is cross-sectional with self-report data. Confirmation of the presence of chronic diseases through medical records was not possible.

## CONCLUSIONS

Based on the results of our study, major chronic diseases such as arthritis/rheumatism, heart disease, high blood pressure, cataracts, and diabetes were found to have a severe impact on HRQL. Urinary incontinence, Alzheimer/other dementia, effects of stroke, cancers, thyroid condition, and back problems had a moderate impact. Food allergy, allergy other than food, asthma, migraine headaches, and other remaining chronic diseases were found to have a relatively mild effect. The HUI scores facilitate the measurement and interpretation of results of health burden and the HRQL for individuals with chronic conditions, and the development of strategies for the prevention and control of chronic diseases. Health burdens also appear to affect older age groups more so than younger age groups. Chronic diseases also appear to have a greater impact on the male quality of life compared to females for diabetes, heart attack, and allergy other than food.

## ACKNOWLEDGMENTS

The authors gratefully acknowledge Mrs. Sylvain Paradis, Éric Jenkins, and Mario Bédard for their positive comments and the information concerned this paper.

## BIOSKETCHES

**Frank Mo, PhD, MD**, Centre for Chronic Disease Prevention and Control, Population and Public Health Branch, Health Canada. E-mail: Frank_Mo@hc-sc.gc.ca Website: http://www.hc-sc.gc.ca/pphb-dgspsp/ccdpc-cpcmc/about_e.html

**Bernard C.K. Choi, PhD, MSc**, Centre for Chronic Disease Prevention and Control, Population and Public Health Branch, Health Canada. Website: http://www.hc-sc.gc.ca/pphb-dgspsp/ccdpc-cpcmc/about_e.html

**Felix C.K. Li, MB BS, FRCPC**, Centre for Chronic Disease Prevention and Control, Population and Public Health Branch, Health Canada. Website: http://www.hc-sc.gc.ca/pphb-dgspsp/ccdpc-cpcmc/about_e.html

**Joav Merrick, MD, DMSc,** is Professor of Child Health and Human Development affiliated with the Zusman Child Development Center, Division of Pediatrics and Community Health at the Ben Gurion University, Beer-Sheva, Israel; the Medical Director of the Division for Mental Retardation, Ministry of Social Affairs, Jerusalem; and the Founder and Director of the National Institute of Child Health and Human Development. He has numerous publications in the field of child and human development, rehabilitation, intellectual disability, disability, health, welfare, abuse, advocacy, quality of life, and prevention. Dr. Merrick received the Peter Sabroe Child Award for outstanding work on behalf of Danish Children in 1985 and the International LEGO-Prize ("The Children's Nobel Prize") for an extraordinary contribution towards improvement in child welfare and well being in 1987. E-mail: jmerrick@internet-zahav.net. Website: www.nichd-israel.com
